# Epigenetic control of cell identity and plasticity

**DOI:** 10.1186/1471-2164-15-S2-O11

**Published:** 2014-04-02

**Authors:** Valerio Orlando

**Affiliations:** 1 Biological and Environmental Sciences and Engineering Division, KAUST Environmental Epigenetics Research Program, 4700 King Abdullah University of Science and Technology, Thuwal, Kingdom of Saudi Arabia

## 

The DNA centered dogma for genetic information and cell identity is now evolving into a much more complex and flexible dimension provided by the discovery of the Epigenome. This comprises those chromosome structural and topological components that complement DNA information and contribute to genome functional organization.

Current concept is that the Epigenome constitutes the dynamic molecular interface allowing the Genome to interact with the Environment. Exploring how the genome interacts with the environment is a key to fully understand cellular and complex organism mechanisms of adaptation and plasticity.

Our work focuses on the role of an essential, specialized group or chromatin associated proteins named Polycomb (PcG) that control maintenance of transcription programs during development and in adult life. In particular PcG proteins exert epigenetic “memory” function by modifying chromosome structures at various levels to maintain gene silencing in particular through cell division. While in the past decade substantial progress was made in understanding PcG mechanisms acting in development and partially during cell cycle, very little is known about their role in adult post-mitotic tissues and more in general the role of the epigenome in adaptation.

To this, we studied the role of PcG in the context of mammalian skeletal muscle cell differentiation. We previously reported specific dynamics of PRC2 proteins in myoblasts and myotubes, in particular the dynamics of PcG Histone H3 K27 Methyl Transferases (HMT), EZH2 and EZH1, the latter apparently replacing for EZH2 in differentiated myotubes. Ezh1 protein, although almost identical to Ezh2, shows a weak H3K27 HMT activity and its primary function remains elusive. Recent ChIPseq studies performed in differentiating muscle cells revealed that Ezh1 associates with active and not repressed regulatory regions to control RNA pol II elongation. Since H3K27 tri-methylation levels are virtually steady in non-cycling myotubes we asked which could be PRC2-EZH1 function in this context. We postulated that the presence of PRC2-EZH1 complex on active promoters, and not on repressed loci, could be explained by a novel role for PcG to control prompt response to specific to stress conditions and silencing of tissue specific active genes in a global coordinated manner.

Data *in vitro* and *in vivo* will be presented in support of our hypothesis showing that in terminally differentiated myotubes PRC2-Ezh1 complex H3K27m3 methyltransferase activity is dynamically regulated in response to specific environmental stimuli and that EZH1 function is required in particular to prevent muscle atrophy. Further, we discovered that this dynamics involves a novel pathway controlling PRC2 complex formation, with potential general implications for mechanisms controlling the ability of somatic cells to respond to adverse environmental conditions via chromatin plasticity.

